# ‘Get Healthy!’ A physical activity and nutrition program for older adults with intellectual disability: pilot study protocol

**DOI:** 10.1186/s40814-018-0333-1

**Published:** 2018-08-25

**Authors:** Carmela Salomon, Jessica Bellamy, Elizabeth Evans, Renae Reid, Michelle Hsu, Scott Teasdale, Julian Trollor

**Affiliations:** 1 Department of Developmental Disability Neuropsychiatry, 34 Botany St, UNSW Sydney, Sydney, 2052 Australia; 2 Department of Exercise Physiology, School of Medical Sciences, Wallace Wurth Level 2, UNSW Sydney, Sydney, 2052 Australia; 3 The Boden Institute of Obesity, Nutrition, Exercise & Eating Disorders, The University of Sydney, Sydney, 2006 Australia; 4 Keeping the Body in Mind Program, South Eastern Sydney Local Health District, 26 Llandaff Street, Bondi Junction, 2022 Australia; 5 School of Psychiatry, UNSW, Hospital Road, Randwick, 2013 Australia

**Keywords:** Intellectual disability, Ageing, Physical activity, Exercise, Nutrition, Protocol, Photographic food record, Accelerometry, Intervention, Health promotion

## Abstract

**Background:**

Older adults with intellectual disability have high rates of lifestyle**-**related illness yet remain poorly engaged in physical activity and nutrition interventions. There is a need to clarify what types of healthy lifestyle interventions are feasible and effective to implement in this population and how outcome measures can best be tracked. This paper describes the pilot feasibility study protocol for implementing a 12-week physical activity and healthy eating program, ‘Get Healthy!’ with older adults with intellectual disability.

**Methods:**

The primary study aims are to assess the feasibility of implementing and monitoring the ‘Get Healthy!’ program with adults with mild to moderate intellectual disability, aged 40 years and over, and their carers. Secondary study aims are to assess the impact of the intervention across the following parametres: body mass index**,** waist circumference**,** cardiovascular fitness**,** physical activity (amount and intensity) and sedentary behaviours**,** resting blood pressure**,** functional strength/capacity**,** dietary intake (energy intake, food group consumption and diet quality)**,** dietary and physical activity knowledge**,** and quality of life. Between 8 and 10 participants in total will be recruited into the 12-week program that will be run in metropolitan NSW, Australia. A combination of objective and subjective measures will be used to assess program feasibility and impact at set timepoints (baseline, mid and end-program).

**Discussion:**

Results from the feasibility pilot will be used to refine the study methodology and ‘Get Healthy!’ program content for future use in a sufficiently powered trial. Findings may be of interest to a broad range of disability and allied health workers engaged in supporting and monitoring healthy lifestyle change in adults with intellectual disability.

**Trial registration:**

ACTRN: ACTRN12618000349246. Registered March 8, 2018- Retrospectively registered, UTN: U1111-1209-3132.

## Background

Modifiable lifestyle-related behaviours contribute to illness [1] and premature mortality [2] in people with intellectual disability (ID). Across the lifespan, people with ID are more likely than aged-matched peers to be overweight or obese [3–5]. They are less likely to reach recommended physical activity levels and are more likely to engage in sedentary behaviours [6–8]. Additionally, people with ID tend to be less knowledgeable about healthy eating recommendations and more likely to consume diets that are high in discretionary foods (high fat, sugar, salt foods, low in essential nutrients), particularly as snacks, and low in core foods including fruits and vegetables [9–11]. The increased cardiometabolic risk associated with such behaviours is compounded in this population by side effects of commonly prescribed psychotropic medications [12, 13].

Healthy lifestyle interventions in the general population are growing in popularity and have been shown to be effective in reducing lifestyle risk [14–16]. However, despite an increased cardiometabolic risk profile, people with ID remain poorly engaged in preventative care programs [17] including healthy lifestyle interventions [18]. Unlike the progressive decline in the absolute number of cardiovascular related deaths in the general population, there has been no similar decline among people with ID [19], suggesting that health promotion messages and interventions have not yet effectively engaged this population. Rates of potentially modifiable morbidities such as diabetes, high blood pressure, hyperlipidaemia, some cancers, dental decay, malnutrition and osteoporosis remain alarmingly high among people with ID [17, 20–22]. As well as having a significant impact on physical wellbeing, experiences such as being overweight or having dental decay can also lower an individual’s self-esteem and sense of psychological wellbeing [23, 24].

Many barriers exist for people with ID who seek to access healthy lifestyle programs to address these risks. These include lack of transport; lack of funding; stigma, a workforce that is insufficiently equipped to tailor interventions to the unique needs of this population; cognitive, behavioural and communication challenges; and a lack of universal design in urban planning that prevents some people with co-morbidities from accessing public exercise areas [1, 25, 26]. Compared to the robust knowledge base detailing healthy lifestyle interventions in the general population, i.e. [27], less is known about the efficacy of lifestyle interventions for people with ID [28]. Recruitment challenges and the additional complexities of obtaining ethics approvals to include people with ID in clinical trials [29, 30] mean that this population has been underrepresented in healthy lifestyle trials to date. Information about how outcome measures can best be tracked across trials are also lacking as many measurement tools have not been validated in this population.

The small number of ID specific physical activity and healthy eating intervention trials reported in the literature to date has resulted in improvements in fitness and psychosocial functioning [31]. Potential for weight loss appears most likely when both physical activity and nutrition needs are addressed simultaneously [32] within a comprehensive health behaviour education program [31]. Making interventions person centred, facilitating carer involvement [10], providing a higher frequency of session and level of support and sufficiently tailored content, design and delivery also appear to improve effectiveness [33]. For example, Melville and colleagues’ [34] intervention that used a combination of behavioural change techniques, carer involvement and physical activity and nutrition-related supports, produced significant decreases in weight, waist circumference and sedentary behaviours for participants with ID.

Results from published ID lifestyle interventions have not been uniformly successful, however, and further research is needed to clarify the type, intensity and delivery mode of interventions that would be most likely to consistently produce positive change in this population [35]. Hamilton and colleagues review of weight loss interventions in this population, for example, find that while most provided evidence for weight loss during active phase, the evidence for long-term weight loss maintenance is less clear [10]. They also note that some interventions appear to produce a smaller degree of weight loss than would be expected in the general population. Other interventions, however, have found that weight loss interventions can be equally effective for people with and without ID [36]. Controlled physical activity trials for adults with ID such as *Walk Well* [33] did not produce any changes from baseline, despite the parent program’s proven efficacy in the general population. The *Steps To Your Health* trial [37] likewise reported no statistically significant difference in mean moderate-vigorous physical activity level or BMI compared to controls. Further studies that robustly measure physical activity levels and dietary intake are needed to clarify the essential components of best practice lifestyle intervention delivery to this population.

Particularly lacking are studies exploring the impact of healthy lifestyle interventions on older people with intellectual disability. Advances in healthcare and public policy have produced significant improvements in longevity for people with ID [38], yet little is known about how health promotion messages and interventions can best be targeted to this growing demographic. Older people with ID experience higher rates of both physical and mental health morbidities than the general population and are vulnerable to premature ageing, often as early as in their forties, as well as stigma, poverty and social isolation [19]. Targeting lifestyle-related behaviours such as physical activity has been identified as potentially the most impactful single intervention to improve health outcomes for people with ID [39], including the ageing population [1].

### Background to the ‘Get Healthy!’ program

**Fig. 1:**
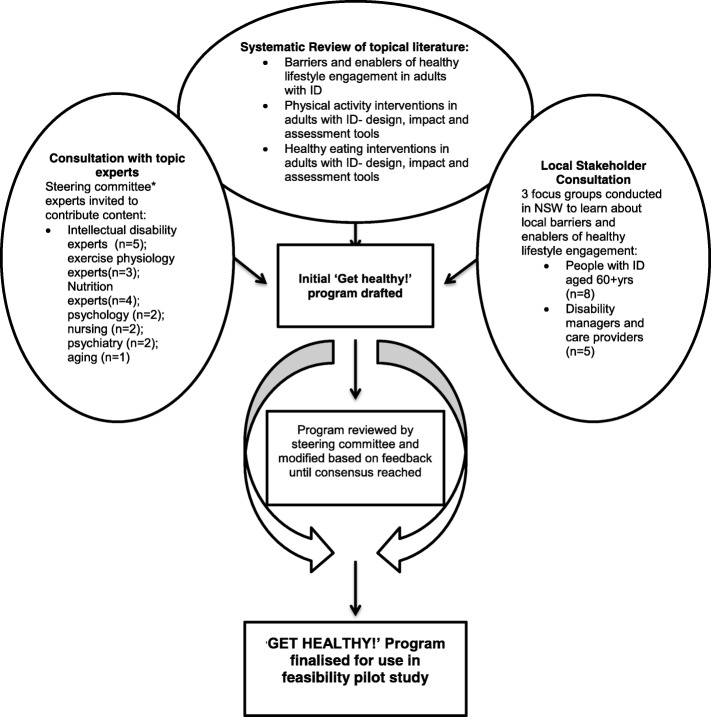
Development of the ‘Get Healthy!’ program. The project steering committee members invited to provide feedback were Prof Julian Trollor, Dr. Carmela Salomon, Dr. Liz Evans, Chris Tzarimas, Dr. Simon Rosenbaum, Jess Bellamy, Dr. Jackie Curtis, Prof. Katherine Samaras, Andrew Watkins, Assoc. Prof Philip Ward, Scott Teasdale, Michelle Hsu and Renae Reid. Additionally, nutrition and exercise physiology student researchers Chan, J., Wang, Q., Heinonen, T., Chub, C., Tripodi, E., Chen, A., Guo, X. and Bartter, J. contributed to the program design

Figure 1 summarises the development process for the ‘Get Healthy! Program, a 12-week healthy lifestyle program designed to support the health promotion needs of older adults with mild to moderate ID. The program provides tailored nutrition and physical activity education as well as guided physical activity sessions in a small group setting. As shown, the structure and content of the program were developed through a combination of expert consultations, systematic literature reviews and stakeholder consultation with managers, carers and people with ID aged 60 years and older. The stakeholder consultation process highlighted the importance of addressing both environmental, person related and organisational barriers when planning a healthy lifestyle intervention. Participants highlighted a preference for group interventions that offered opportunities for social interactions, while carers emphasised the importance of ensuring interventions are adequately resourced to meet the complex needs of clients. Detailed findings from the stakeholder consultations are reported elsewhere [40].

A summary of physical activity and nutrition content, and session structure, as well as required equipment, is provided in Table 1. Participants in the ‘Get Healthy!’ program engage in a minimum of three contact hours each week, for a continuous 12-week period. Contact hours are made up of two 1-h physical activity sessions, held on non-consecutive days, and 1 h per week of guided nutrition information and support. In order to decrease participant time and transportation burden, we will run the weekly nutrition session immediately before or after one of the exercise sessions. Participants will therefore only need to attend the program on two out of 7 days per week. The physical activity component of the program is delivered by an accredited exercise physiologist, and the nutrition-related component of the program is delivered by an Accredited Practicing Dietitian. Both of these program leaders have previous experience running interventions for people with intellectual disability. Intervention intensity and advice is tailored to the individual needs of each participant; however, the program is ideally delivered in a small group setting (three to six people per group). The program can be run in a number of settings; however, access to specialised exercise and monitoring equipment is required for pre- and post-testing as well as some of the physical activity sessions*.* In order to support sustained behaviour change post-intervention, the exercise physiologist will also provide examples throughout the program of accessible and low-cost ways to increase physical activity without specialised equipment.

**Table 1:** ‘Get healthy!’ curriculum content

Component	Time commitment	Topics	Resources required^a^
Nutrition	12-week program consisting of the following: One face-to-face 60 min session per week Plus additional follow-up phone calls/prompts during food intake monitoring periods. Phone calls will be made either directly to the participant or to their carer depending on the person’s level of independence.	The style of presentation will be tailored to the abilities of participants. The nutrition *education* component will be presented to all participants and will cover the following topics: • The five food groups • Discretionary foods and healthy snacks • Healthy drinks • Portion size and mindful eating • Eating out choices • Shopping/eating out tour	Equipment: • Scale • Stadiometre • Cameras for each participant • 24-h proxy food recall form • Folder for each participant to keep their handouts • White board • Computer and projector Handouts: • Instructions to complete 3-day digital photography food record • 12-week challenge checklist • ID-adapted Australian dietary guidelines • Nutritional goals and barriers • Weight and BMI • Information about discretionary foods, fruits and vegetables. • Information about the grain, meat and dairy food groups • Healthy snack choices • Are you drinking enough water? • Drink ideas • Healthy plate model, hunger scale and mindful eating • Healthier eating/takeaway options • What to think about when eating out • Healthy Eating Graduate certificate
Physical activity	12-week program consisting of the following: Two 60-min face-to face sessions per week on non-consecutive days. Breakdown of components within each session: - 10% didactic information - 40% aerobic exercise - 30% strength-based exercise - 20% balance-based exercise	The physical activity *education* component will cover the following topics: • What it means to be healthy • Consequences of obesity • Physical activity and screen time guidelines • Appropriate goal setting • Planning for maintenance and self-management • Barriers to Physical activity and how to address them The physical activity *practical* component will be comprised of a combination of aerobic and strength- and balance-based exercise. Types of exercises and exercise intensity to be tailored to abilities and interests of each participant but may include activities such as: cycling on a stationary bike, walking, supported row, horizontal squat, stair climbing, tandem, body weight exercises such as wall push-ups, light dumbbell work.	Equipment: - GTX3+ accelerometres for each participant - Blood pressure Sphygmomanometre and stethoscope - Scale - Stadiometre - Measuring tape - Stationary bikes (including a Monark resistance-based ergometre) - Heart rate monitors - Stop watch - Trundle wheel - Medicine balls - Parallel bars - Stairs - Boxing equipment - Chairs - Exercise mats - Cones - Dumbbells - Horizontal squat machine - Supported row machine - Rower Handouts: - ‘Goal setting’ - ‘Counting our steps’

^a^Handouts listed here are for participants with mild to moderate intellectual disability. They may be modified further depending on the needs of individual participants. Carers participating in Program A will be offered the same information in handouts tailored to their level of health literacy

**Fig. 2:**
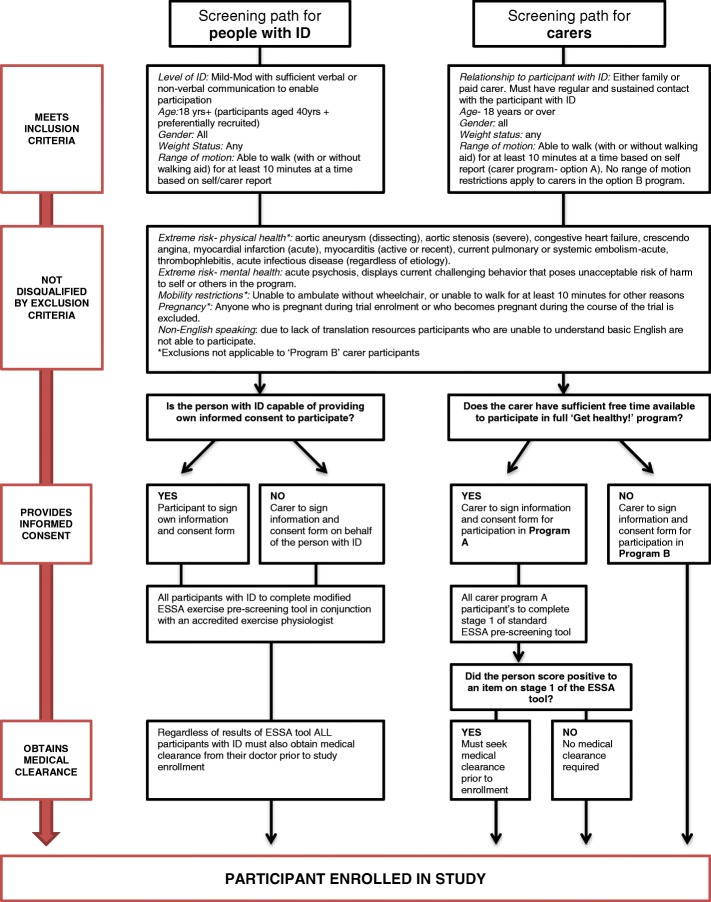
Participant screening procedure

Based on findings from the literature that the physical activity and nutrition behaviours and beliefs of carers strongly influence those of people with ID [41] and that carers can effectively support healthy lifestyle change for adults with ID [42] the ‘Get Healthy!’ program includes two parallel protocols for engaging carers as co-participants. Carer protocol A allows carers to participate for the same number of sessions as participants with ID and covers the same content. Carer A participant sessions will be run separately to the sessions for people with ID so as to more effectively tailor teaching resources to the different learning needs of both groups. Carer protocol B allows carers who lack time or capacity to engage in the full program to access elements of the program in a more flexible way. This may include attending exercise or nutrition sessions with the participant with ID when able, as well as receiving written information about healthy eating and physical activity. All pathways to participation are summarised in Fig. 2.

### Aim

This is a pilot study designed to answer the following primary research questions:

i. Is the ‘Get Healthy!’ program feasible to implement in participants with mild-moderate ID, aged 40 years and over, and their carers?
ii. Are the instruments and procedures being used to collect clinical outcome measurement data feasible to administer to participants with mild-moderate ID, aged 40 years and over?

The secondary aim of the study is to determine, in a small sample, if any pre-post program changes occur across the following clinical outcome measures: body mass index, waist circumference, cardiovascular fitness, physical activity (amount and intensity) and sedentary behaviours, resting blood pressure, functional strength/capacity, dietary intake (energy intake, food group consumption and diet quality), dietary and physical activity knowledge, and quality of life.

## Methods

### Design

This is a single intervention pilot feasibility study.

### Study population

Inclusion/exclusion criteria: Participants with mild to moderate intellectual disability and their carers will be recruited into the study. Level of ID will be determined based on the report of referring disability agencies or primary caregivers. In order to test the feasibility of the ‘Get Healthy!’ program for older adults with ID, participants aged 40 years and over will be preferentially recruited. Participants deemed to be at extreme psychiatric, behavioural or physical health risk, and participants with an insufficient range of motion to participate in the physical activity component of the program will be excluded from the study. Full inclusion and exclusion criteria for both the person with ID and carer groups are outlined in Fig. 2.

Sample size: As this is primarily a feasibility study, it is not powered to demonstrate statistical significance. Between 8 and 10 adults with intellectual disability and carers in total will be recruited using convenience sampling. This sample size and recruitment methodology is based on the research teams’ previous recruiting experience with this difficult to access population. Limiting the study sample size will also allow us to engage with each participant’s progress in a detailed fashion. We will seek comprehensive feedback from each individual in order to refine the program for future use.

Recruitment and screening: Participants will be recruited from a defined target area in metropolitan NSW, Australia. Intellectual disability service and housing providers, partner organisations and peak bodies within the target area will be approached to advertise the study. Potential participants meeting inclusion criteria will undertake a multi-stage screening procedure prior to enrolment. The screening pathway is outlined in detail in Fig. 2 and includes the following:

I. Obtaining informed consent from the person or proxy consent from their next of kin or guardian as required by law. Capacity to provide consent will be based on a combination of carer/disability service provider feedback and research team assessment.
II. Completion of the Exercise and Sports Science Australia (ESSA) adult pre-exercise screening tool [43] (carer participants—protocol A). Participants with ID also to complete, with some modifications including tool to be completed in conjunction with qualified exercise physiologist and carer where relevant, language in tool has been simplified to increase accessibility, addition of a screening question for people with Down’s syndrome related to spinal cord compression or atlantoaxial instability, and all participants with ID to be referred for medical clearance irrespective of screening tool results.
III. Obtaining medical clearance to participate (all participants with ID, as well as all carer program A participants with a positive score on stage 1 of the ESSA screening tool). The research team will offer to reimburse costs relating to attending the medical clearance appointment.

If we have a greater number of people applying for the trial than we have spaces available, we will consider factors such as gender (with the aim of ensuring both men and women are represented in the sample) and age (preferentially recruiting participants who fall into our target range of 40 years and over).

### Data collection timeline

Table 2 summarises the data collection timeline for all outcome measures.

**Table 2:** Outcome measures and data collection points

Outcome measure		Time point (weeks)													
		0 Baseline	1	2	3	4	5	6	7	8	9	10	11	12	13 Completion
Attendance and participation		X	X	X	X	X	X	X	X	X	X	X	X	X	X
Body mass index	Height	X													
	Weight	X						X							X
Waist circumference		X						X							X
Blood pressure		X						X							X
Cardiovascular fitness YMCA sub-maximal ergometre test − 12-min duration	minutes/stages performed	X						X							X
	Peak HR (%APMHR)	X						X							X
	Peak workload achieved	X						X							X
Physical activity level and sedentary behaviour	Self/proxy report	X													X
	Actigraph data	X													X
Physical strength tests	30-s modified push up test	X													X
	Medicine ball throw/chest pass	X													X
	10RM testing	X													X
	30-s sit to stand test	X													X
Quality of life		X													X
Dietary intake assessment	3-day photographic food record	X													X
	Proxy-assisted 24-h recall	X													X
Nutrition and physical activity knowledge		X													X

### Outcome measures

The feasibility indicators described below are the primary outcome measures being assessed in this pilot. Clinical outcomes will be explored as secondary outcome measures.

### Feasibility outcomes

Participation rates: Individual participation in each week’s nutrition session and each physical activity sessions will be scored:

- 0 = Did not attend
- 1 = Attended but only participated minimally. For example, attempted < 50% of activities, did not join in group discussions
- 2 = Attended and participated moderately well to very well. For example, engaged in > 50% of activities, was part of group discussions.

At the completion of the intervention these scores will be totalled and participants will be categorised into high (100–75% score), medium (50–74% score) or low (< 50% score) participation groups for the purposes of analysis.

Drop-out rates and reasons: Drop-out rates and reasons will be recorded to help determine the acceptability of the program to participants.

Acceptability of data collection measures and procedures: The distribution of missing data within each measure will help to determine the acceptability of measures to program participants. Exit interviews will also be conducted with all participants to capture qualitative data on the acceptability of the program and data collection measures for individual participants. Exit interview schedules have been designed with accessible language to enable participation. All interviewers will have had previous experience leading interviews or focus groups with people with ID.

Adverse outcomes: Participants who develop any physical injury or other emerging health issue during the trial will be referred back to their GP for review and clearance prior to continuing. Participants displaying psychological distress will be referred to relevant mental health support services. All adverse physical and psychological outcomes will be recorded and reported in subsequent publications.

Benchmark results indicating a definitive larger scale trial may be feasible will include the following:

- Drop-out rate of < 70%
- A majority of participants (> 60%) achieving ‘high’ or ‘medium’ participation scores
- Positive qualitative feedback from > 80% of participants regarding their experience of undertaking the program
- The feasibility of each individual outcome measure will be assessed separately. If less than half of participants were able to complete the outcome measure the research team will review in detail reasons for failure to implement the measure. Based on this review, we will either modify how the measure is implemented in the larger trial (for example, providing participants with additional prompts and support to complete a measure), or replace the outcome measure if it appears impractical to implement in our target population.

### Clinical outcomes

Body mass index (BMI) will be calculated using the formula BMI = weight/height (kg/m^2^). Participants will be weighed to the nearest 100 g. Height in metres will be measured to the nearest 1 mm. All participants will be requested to remove their shoes and heavy jackets/overcoats prior to data collection.

Waist circumference (WC) will be measured at the midpoint between the iliac crest and the lowest rib, in full expiration, to the nearest 0.1 cm while the person is standing.

Cardiovascular fitness (CV fitness) will be assessed using the YMCA sub-maximal ergometre test (12 min duration): number of minutes/stages performed, peak HR (% of APMHR), peak workload achieved and predicted VO_2_ peak (ml/kg/min) if appropriate.

Physical strength will be measured using 30-s modified push up test, medicine ball throw/chest pass, 10RM strength testing and 30 s Sit to Stand test.

Blood pressure will be measured using a sphygmomanometre while the participant is seated and has rested for least 5 min prior to data collection. Blood pressure is taken in both the right and left arms. Forearm will be placed at mid-sternal level during the procedure.

Engagement in physical activity (PA) and amount of sedentary behaviour (SB): *Objective PA and SB data* will be collected using a waist-based GTX3 actigraph accelerometre worn for a period of 5 days in each data collection period. Participants will be encouraged to wear the actigraph at all times during these days, apart from when they shower. Information about steps, and percentage of sedentary behaviour and light, moderate-vigorous PA will be collected.

In order to make our results comparable with earlier studies in adult ID populations, we will use the actigraph protocol outlined in Harris et al. [44] that involves the following:

- A minimum requirement of at least 6 h of data on at least three of the five wear days
- Accelerometres to record activity over 15-s intervals, with activity counts of four consecutive epochs summed to give activity counts per minute (cpm). Four categories of activity intensity defined for data analysis:
  • Sedentary behaviour 0–499 cpm
  • Light intensity activity 500–1951 cpm
  • Moderate intensity activity 1952–5724 cpm
  • Vigorous intensity activity greater than 5725 cpm
- The accelerometre data will then be used to calculate the mean time (minutes) and the percentage time per day, spent in each level of activity.

Detailed information about the validity and reliability of objective measurements of physical activity and sedentary behaviour in adults with ID are lacking. However, actigraph accelerometres are the most commonly used objective measurement tool in the adult ID literature to date. At least nine previous studies with adults with ID [33, 34, 37, 44–49] have used this methodology, either alone or in combination with subjective measures. The majority of these studies used a sedentary behaviour cut-off point of < 500 counts per minute in view of recent findings that adults with ID spend more energy when engaged in sedentary activities than the general population [50, 51].

*Subjective PA and sedentary behaviour data* will be collected using the International Physical Activity Questionnaire-proxy respondent (IPAQ-pr) proxy report [52]. Information will be collected from a carer with input from the participant with ID where possible. The purpose of the subjective questionnaire is to elicit more detailed information about the types of PA and sedentary behaviours the person is engaged in. For participants who are unable to tolerate the actigraph, this questionnaire may also provide a useful approximation of changes in PA and SB levels across the intervention. In cases where both measures are successfully collected, we will link the actigraph and IPAQ-pr data to explore concurrence of findings across measures.

The original IPAQ measure was a 7-day self-report recall questionnaire and was not developed for people with ID. This instrument has good psychometric properties [51]. The IPAQ-pr see [52] changed the original IPAQ from a 7-day recall questionnaire to an activity diary to be completed by carers. The IPAQ-pr was found to be a reliable and valid measure for categorising whether participants with ID met recommendations for physical activity levels when completed by a parent or support staff of the adult with ID [52].

Dietary intake (energy intake, food group consumption and diet quality) will be measured using the following:

- Three-day photographic food record and a proxy-assisted 24-h recall, interpreted and analysed by two Accredited Practicing Dietitians using Foodworks® (version 9) nutrition analysis software (Xyris Software, 2018) and cross checked for inter-rater reliability.
- Calculation of Healthy Eating Index for Australian Adults (HEIFA) to measure diet quality, conducted by at least one Accredited Practising Dietitian.

The 3-day photographic food record is a validated method for assessment of food intake in adults with ID [32, 53–55]. The 3-day photographic food record method was compared to direct observation by an investigator in 18 adults with ID over a 1-day period with an intra-class correlation of 0.84, *p* < 0.001 [54]. This method was selected as studies suggest a 3-day weighed food record (WFR) may be impractical for people with ID due to cognitive impairments and difficulty in measuring portion sizes. Proxy-assisted food records may also be impractical with studies reporting inaccurate measurements and reduced fidelity when burden is placed on the carer for data collection. A 3-day digital photography gives participants independence and allows food consumed away from carer to be easily captured [54]. Reminder messages will be provided by investigators to maximise compliance. The Accredited Practising Dietitians will clarify photographic records with participants and parents/carers prior to analysis, utilising a food checklist to minimise foods missed from the photographic food record.

Foodworks® nutritional analysis program (version 9) provides a comprehensive breakdown of energy, macro- and micronutrient, fat subgroups, added sugar, fibre and food group intakes. Data from this program will be entered into a pre-developed Microsoft Excel spread sheet to calculate the Healthy Eating Index for Australian Adults (HEIFA-2013) score. The HEIFA-2013 is a singular score based how close dietary intake is to the current Australian Dietary Guidelines [56]. The tool is validated in young adults 18–34 years. The tool has not been validated in older adults or ID population. To our knowledge, diet quality has not been previously studied in older adults with ID, and therefore, this study could provide a unique set of data. A study in Queensland, Australia, is applying a 4-day WFR and the HEIFA to assess the diet quality of individuals with a disability undergoing a sporting intervention (unpublished data).

Nutrition and physical activity knowledge will be assessed using the Nutrition and Activity Knowledge Scale for Use with People with an Intellectual Disability (NAKS) questionnaire. This questionnaire has been reported to have good psychometric properties in adults with ID. For detailed information about the validity and reliability of this measures see Illingworth et al. [57]. Previous studies examining nutrition and physical activity knowledge in populations with ID have also reported successfully using this measure [58–60].

Quality of life will be measured using the Personal Wellbeing Index - Intellectual Disability (PWI-ID) [61]. This index is administered to the person as an interview and covers the following domains: standard of living, health, life achievement, personal relationships, community-connectedness and future security. The adult version of the measure has been reported to have good reliability and validity when used with adults with ID [62].

Data analysis plan: Quantitative data will be analysed using the Statistical Package for Social Science (SPSS). Given the small sample size, primary analysis will focus on descriptive statistics and confidence interval estimations for outcomes of interest. While this trial is not powered to detect statistically significant differences, we will use paired *t* tests in an exploratory fashion to analyse normally distributed continuous variable data collected at two timepoints only (baseline and 13 weeks). The nonparametric Wilcoxon signed-rank test will be used in cases where data are not normally distributed. To explore any group effects of the intervention when there are two timepoints, we will calculate the difference between completion and baseline timepoints for each measure and then compare the mean of the differences using *t* tests. Regression modelling adjusting for clustering will be used to analyse data collected at three or more timepoints. Appropriate imputative techniques will be used to account for any missing data points where appropriate. We will also analyse the impact of gender on feasibility and clinical outcomes. This analysis will be exploratory in nature in view of the small sample size. Qualitative data collected from exit interviews will be digitally recorded and transcribed verbatim. Transcribed data will be thematically analysed to identify program strengths, weaknesses, suggestions for improvement, and overall perceived impact. The software program NVivo (version 11.0.0) will be used to support organisation of qualitative data.

## Discussion

This study described here aims to establish the feasibility and impact of running the 12-week physical activity and healthy eating program, ‘Get Healthy!’ with a group of participants with mild-moderate ID and their carers. Detailed data regarding participants’ engagement in physical activity, nutritional intake, and personal wellbeing will be collected. The inclusion of the exit interview within the study protocol will help to identify potential barriers to the success of the program. Our goal is to recruit participants with ID aged 40 years and over; however, if we are unable to recruit a sufficient number of participants in this age range, younger adult participants (aged 18 years and over) may also need to be included. While there is a clear need to conduct further research about healthy lifestyle interventions for people with more severe-profound levels of ID, our current intervention is limited to participants with mild-moderate ID and their carers. The support needs, teaching formats and resources required to engage people with more severe-profound levels of ID in a healthy lifestyle intervention are significantly different to those of people with mild-moderate ID. It was considered beyond the scope of the study to develop and evaluate two different intervention packages. Results of this study will be used to refine the methodology and program content for future use in a sufficiently powered efficacy trial with longer-term follow-up.

In the context of the growing number of people with ID surviving into older age, it is imperative that we learn more about how to extend quality of life and other health outcomes for this demographic [38]. Improving the engagement of people with ID in healthy lifestyle interventions has been identified as an important strategy for decreasing the significant health disparities faced by this population [1]. This feasibility study will contribute to our knowledge of the most appropriate types of interventions and outcome measures to promote a healthy lifestyle in this high-risk group.

## Abbreviations

BMI: Body mass index
CV Fitness: Cardiovascular fitness
ESSA: Exercise and Sports Science Australia
HEIFA: Healthy Eating Index for Australian Adults
ID: Intellectual disability
IPAQ-pr: International Physical Activity Questionnaire-proxy respondent
NAKS: Nutrition and Activity Knowledge Scale for Use with People with an Intellectual Disability
PWI-ID: Personal Wellbeing Index - Intellectual Disability
WC: Waist circumference
WFR: Weighed food record
